# Statistical Framework for Identifying Differences in Similar Mass Spectra: Expanding Possibilities for Isomer Identification

**DOI:** 10.1021/acs.analchem.3c00495

**Published:** 2023-04-17

**Authors:** Hoi-Ting Wu, Dylan L. Riggs, Yana A. Lyon, Ryan R. Julian

**Affiliations:** Department of Chemistry, University of California, Riverside, California 92521, United States

## Abstract

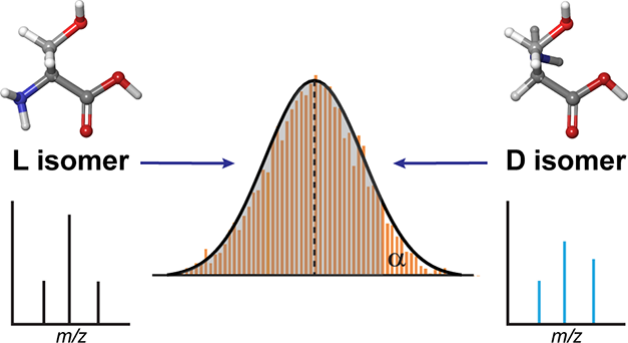

Isomeric molecules are important analytes in many biological and chemical arenas, yet their similarity poses challenges for many analytical methods, including mass spectrometry (MS). Tandem-MS provides significantly more information about isomers than intact mass analysis, but highly similar fragmentation patterns are common and include cases where no unique *m*/*z* peaks are generated between isomeric pairs. However, even in such situations, differences in peak intensity can exist and potentially contain additional information. Herein, we present a framework for comparing mass spectra that differ only in terms of peak intensity and include calculation of a statistical probability that the spectra derive from different analytes. This framework allows for confident identification of peptide isomers by collision-induced dissociation, higher-energy collisional dissociation, electron-transfer dissociation, and radical-directed dissociation. The method successfully identified many types of isomers including various d/l amino acid substitutions, Leu/Ile, and Asp/IsoAsp. The method can accommodate a wide range of changes in instrumental settings including source voltages, isolation widths, and resolution without influencing the analysis. It is shown that quantification of the composition of isomeric mixtures can be enabled with calibration curves, which were found to be highly linear and reproducible. The analysis can be implemented with data collected by either direct infusion or liquid-chromatography MS. Although this framework is presented in the context of isomer characterization, it should also prove useful in many other contexts where similar mass spectra are generated.

## Introduction

Mass spectrometry (MS) is an immensely powerful technique capable of providing molecular-level information on species ranging from small organic molecules to intact viruses.^1,2^ Despite this range of influence, an inherent limitation of MS experiments is that analytes must differ in mass to be distinguished. For the vast majority of analytes, this does not present a problem, but many important biological molecules (and many pharmaceutical molecules that interact with them) can adopt multiple isomeric forms with vastly different biological activities.^3^ Such isomers exist for proteins,^4−6^ glycans,^7^ lipids,^8^ metabolites,^9,10^ and nucleotides.^11^ Due to their biological importance and difficulty with regard to characterization, considerable effort has been exerted to develop methods for isomer analysis.^12−17^

**Scheme 1:**
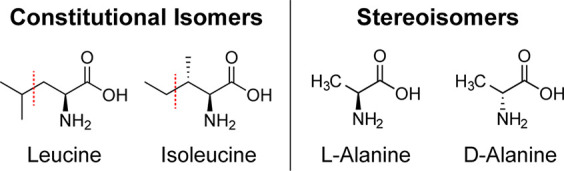
Illustration of Differences in Fragmentation between Classes of Isomers^a^ Constitutional isomers can have unique mass fragments (indicated by a dashed line), whereas chiral stereoisomers cannot.

However, evaluation of isomeric molecules, which share identical exact masses, has historically been difficult to achieve by MS. In fact, it has been uttered at more than one conference that “isomers cannot be analyzed by MS”. This statement is misleading, although it is true that meaningful analysis of isomers requires fragmentation, or MS/MS experiments, to enable characterization. For constitutional isomers and cis/trans double bonds, unique product ions that definitively distinguish specific isomers can be generated by fragmentation, although whether such fragments *will be* generated is not guaranteed and depends on numerous factors.^18,19^ For example, Leucine/Illeucine isomers are not typically distinguishable by proton-initiated fragmentation because the respective side chains do not produce any fragments.^20^ However, for chiral stereoisomers, unique mass fragments simply do not exist regardless of which bonds are broken, meaning that differences between spectra can only exist if there are variations in fragment intensities. Illustrative examples of simple isomers in each class are shown in Scheme 1. In many instances, MS/MS spectra derived from stereoisomeric molecules appear to be very similar.^21,22^ This shortcoming of common collision- or electron-based techniques provided impetus for the development of radical-directed dissociation (RDD), which is a particularly structure-sensitive MS/MS method that often yields highly distinct spectra for isomeric pairs.^23−25^

To quantify differences between spectra obtained from isomeric compounds, we and others have used the *R*-value method.^26−29^ The *R*-value is a ratio that is derived from the peaks that change most between two spectra while ignoring the rest. Although *R*-values can be used to reliably identify isomers with appropriate parameter selection and after calibration on many standard samples, the statistical certainty of the analysis remains unclear and the majority of the data is not included in the evaluation. Many alternative methods for comparing spectral similarity have also been previously described.^30−36^ For example, a common method often employed in proteomics is calculation of the dot product or cosine similarity between two mass spectra.^37−39^ This approach considers all fragment ion masses and intensities, thus increasing the portion of data being utilized relative to the *R*-value method. However, the calculation of the dot product does not take into account the inherent experimental factors that cause variation in ion intensity between mass spectra (as discussed below), which makes it difficult to ascribe an expectation outcome for calculation of the dot product.

In the present work, we introduce a data processing workflow that measures differences between mass spectra. Importantly, a statistical threshold is established to determine if variations in signal intensities exceed the expected variation inherent to repeated analysis. Using this approach, we demonstrate that peptide isomers can be easily distinguished from each other using any commonly available fragmentation method. Although changes in MS/MS spectra between isomers may appear to be subtle, they are reproducibly generated at much higher levels than in the typical variation observed within replicate experiments examining the same peptide. The method is compatible with and can be implemented for the analysis of data derived from LC–MS (liquid-chromatography) experiments, as illustrated by examination of a tryptic digest of human eye lens lysate.

## Experimental Section

### Materials

Organic solvents and reagents were purchased from Fisher Scientific and Sigma-Aldrich and were used without further purification. FMOC-protected amino acids and Wang resins were purchased from Anaspec, Inc. or Chem-Impex International.

### Peptide Synthesis

Peptides were manually synthesized following an accelerated FMOC-protected solid-phase peptide synthesis protocol.^40^ Following synthesis, peptides were stored frozen at −20 °C in 50/50 acetonitrile/water (v/v). Radical precursor peptides were prepared using a previously published method.^25^

### MS Analysis

All peptides were prepared in 50/50 acetonitrile/water (v/v) + 0.1% formic acid with a final concentration of 10 μM. Peptides were analyzed on a Thermo Fisher Scientific Orbitrap Fusion Lumos Tribrid Mass Spectrometer equipped with a 266 nm Nd:YAG laser (Crylas, Berlin, Germany) using direct infusion with a flow rate of 3 μL/min. The capillary temperature, RF voltage, resolution, and spray voltage were set to 275 °C, 50–150%, 30 or 60 k, and 2.8–3.5 kV, respectively. Each series of peptide isomers was examined under the same MS parameters and fragmented by the same fragmentation energy and isolation window width using collision-induced dissociation (CID), higher-energy collisional dissociation (HCD), electron transfer dissociation (ETD), or RDD. After the spray was stabilized with a relative standard deviation (RSD) < 15% for the total ion count, 100 scans were collected for all experiments. The most abundant fragment ions were extracted with in-house software. Statistical analyses were performed with Excel.

### LC–MS/MS Analysis

β-Amyloid (1–9) LC–MS data was collected on a Thermo Fisher Scientific Orbitrap Velos Pro coupled with an Agilent 1100 binary pump using a gradient of solvent A (water with 0.1% FA) and B (methanol with 0.1% FA) with a previously published method.^4^ Purified β-amyloid (1–9) peptide isomers were prepared in water with 0.1% FA and separated on a Thermo BetaBasic C18 3 μm 150 × 2.1 mm column with a standard ionization source. The LC gradient was set to 1% B for 5 min followed by 1% B to 20% B over 45 min with a flow rate of 0.2 mL/min. The LC/MS–MS data for the human eye lens was collected using a Thermo Fisher Easy nLC II using parameters as specified previously.^41^

### Normal Data and Non-Parametric Analysis

The results shown below all derive from one-sample *t*-tests, which assume data normality. In reality, the data are not normal in all instances, but if the sample size is high and the *p*-value is very low, predictions will generally be valid. To verify this prediction, non-parametric analysis was performed and found to agree with the simple *t*-test shown below in every case.

## Results and Discussion

**Figure 1:**
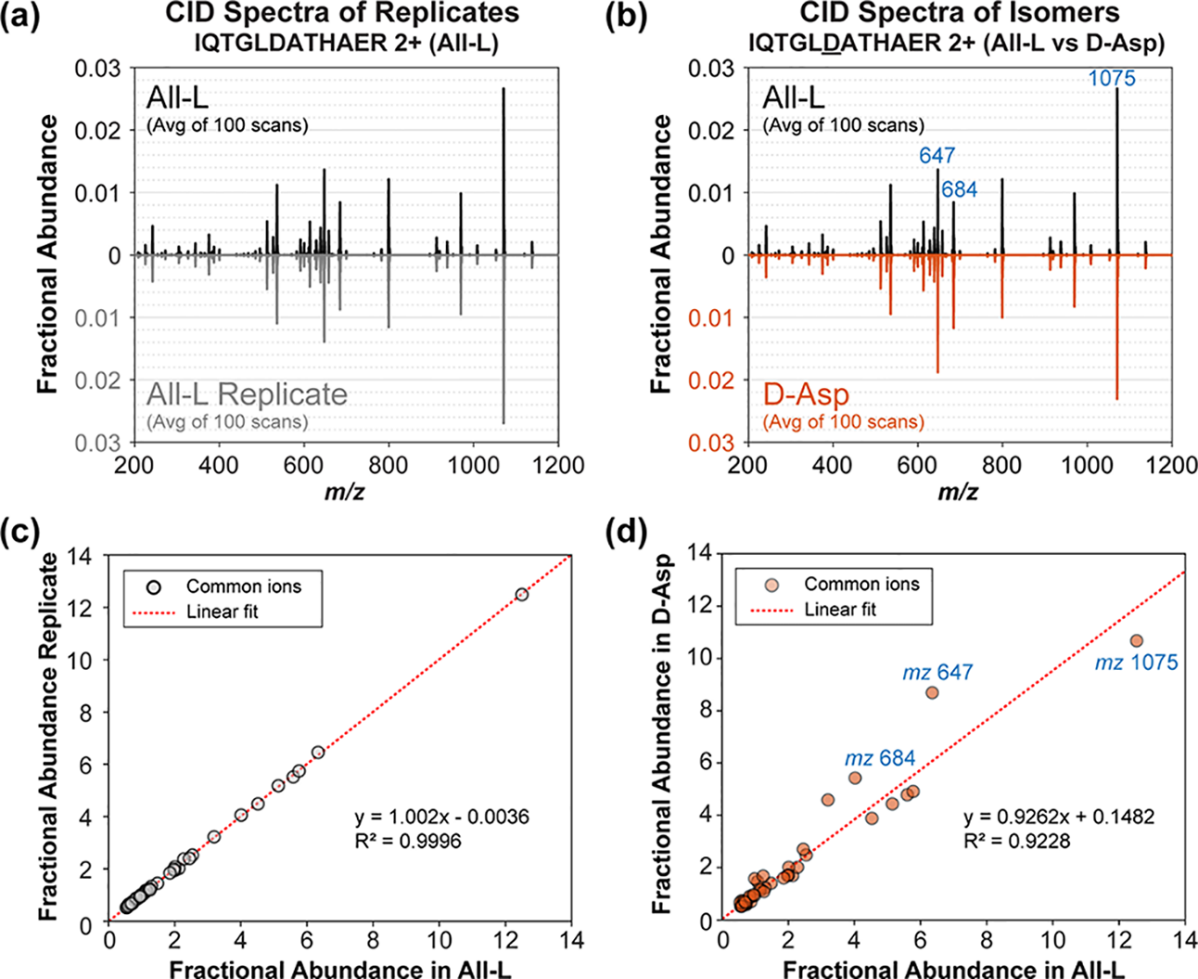
CID mass spectra for IQTGLDATHAER 2+ plotted by fractional abundance in butterfly format for easy comparison. (a) Replicate CID spectra of IQTGLDATHAER (All-l) in black and gray are almost identical. (b) CID spectra of IQTGLDATHAER (All-l) in black and IQTGLDATHAER (d-Asp) are noticeably less similar (for example, the ordering of the 3rd/4th/5th most intense peaks changes). (c) Fractional abundance of common ions in both IQTGLDATHAER (All-l) replicates (gray circle) is highly correlated and yields a slope with a linear fit (red dotted line) close to 1. (d) Plotting fractional abundance of common ions in IQTGLDATHAER L/D-Asp isomers (orange circle) reveals those that differ most (labeled in blue).

To apply statistical probability to the comparison of mass spectra, expected values must be established against which a null hypothesis can be tested. For example, if the same sample were examined by MS/MS twice on the same instrument under identical conditions, we could reasonably expect the fragmentation spectra to be quite similar, with differences being caused by random fluctuations associated with instrument performance, such as source stability, ion transfer efficiency, detection efficiency, etc. A representative example of replicate data is illustrated in Figure 1a for the CID spectrum of [IQTGLDATHAER+2H]^2+^ where the replicate is plotted downward at identical *m*/*z* for straightforward reference. By visual inspection, there are no easily observable variations between the spectra in Figure 1a. To quantitatively evaluate variations, we could take the difference in intensity between each peak. However, even this simple calculation requires some consideration. For example, relative peak ratios are much more reproducible than raw ion counts due to fluctuations in the ionization source. However, subtraction of relative intensities between mass spectra is problematic if the base peak differs between spectra (which can occur for comparison of isomers). Fortunately, this issue is easily resolved by calculating the fractional abundance of each peak (where fractional abundance = [peak height]/[sum of all peak heights]). Plotting the fractional abundances from each peak in the replicate spectra in Figure 1a yields the scatterplot shown in Figure 1c, which illustrates that the replicate datasets are highly correlated. To further quantify differences, the fractional abundances for dataset 1 could be subtracted from the fractional abundances for dataset 2. With ideal instrument performance, the differences between replicates examining the same sample in back-to-back experiments would all be equal to zero. In reality, the magnitudes of these differences will be small, with some negative and some positive, yielding an average value near zero. Importantly, this expected value (nearly zero) is the same for the difference between each pair of peaks in the spectrum, making each fragment an independent variable (or nearly independent). The simplest explanation for why differences are not entirely independent is that the total ion population analyzed by a mass spectrometer under ideal conditions is a fairly consistent number. Therefore, if fractional abundance is lost in one fragmentation channel, it typically arises in another (if the differences are not due to random fluctuations). Put another way, the total ion abundance in MS/MS spectra bears resemblance to a zero-sum game, although this behavior should not be considered or expected to be ideal. Indeed, these considerations are entirely consistent with the framework of unimolecular fragmentation provided by RRKM theory, i.e., the population of different dissociation channels is dictated by the relevant transition states (within the inherent error of the experiment).^42^

In Figure 1b, corresponding CID spectra for two isomeric versions of the same sequence are shown (IQTGLDATHAER with either l-aspartic acid or d-aspartic acid). The spectra are still quite similar, but visual inspection reveals some differences in the intensity for select fragment ions. The differences are again more apparent in the scatterplot shown in Figure 1d. If the differences in fractional abundance are similarly calculated as described for replicate experiments above, a greater variation in fractional abundance differences would be observed for some of the fragments where the isomerized residues exert sufficient influence on the relevant transition states. However, the average value of all differences may still be close to zero because negative differences in one channel can be offset by roughly equivalent positive differences in one or more other channels due to the pseudo-zero-sum game attributes described above.

**Figure 2:**
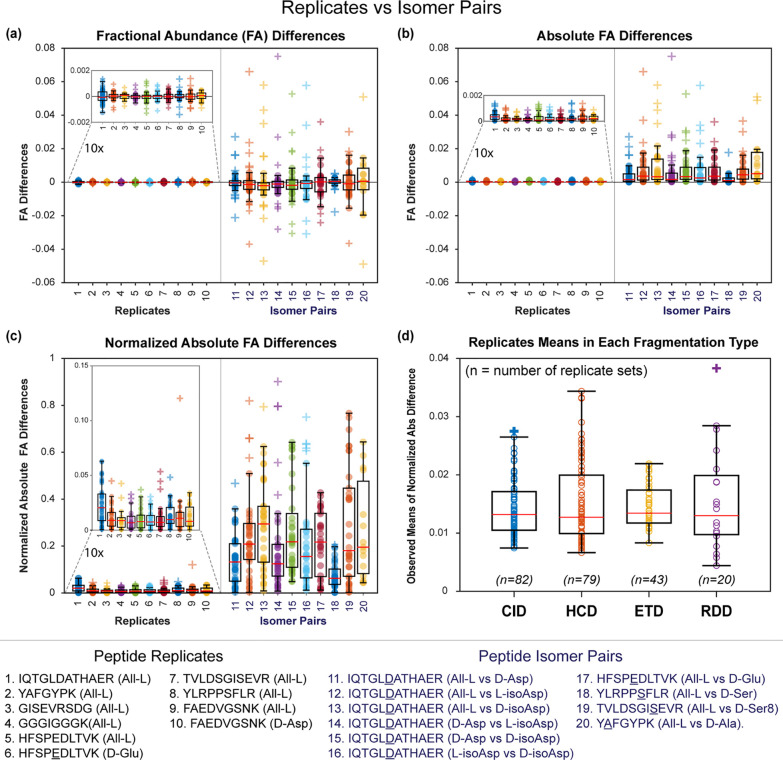
Comparison of [M + 2H]^2+^ CID data for replicates of the same peptide versus isomeric pairs for a series of 10 sequences. (a) Fractional abundance (FA) differences scatter above and below zero for both datasets. (b) Absolute fractional abundance differences hover above zero for replicate data but are significantly larger for isomers. (c) Normalized absolute fractional abundance differences account for changes in peak size. (d) Mean of normalized fractional abundance between replicates plotted for each fragmentation method. Data points are denoted with circles, with plus signs to represent outliers.

To evaluate this model more broadly, we calculated the differences in CID spectra for a series of replicates and isomers. Each data series in Figure 2 represents the cumulative differences between replicate analyses of 10 peptides (series 1–10) and differences between single analyses of the same 10 sequences in two isomeric forms (series 11–20). Individual data points within each series indicate the difference in fractional abundance of a given fragment between the two spectra. The results are displayed in a box plot format in Figure 2a with all contributing data points explicitly shown. The data scatter roughly symmetrically, with similar numbers of peaks either gaining or losing fractional abundance for both isomers and replicates. Furthermore, the average values are close to zero in both cases. This behavior complicates statistical comparison of replicate versus isomeric data sets because both systems trend toward average values near zero, with the primary difference being that larger deviations are observed for comparison of isomeric spectra. It is therefore not optimal to test the statistical likelihood that means are different when the datasets are expected to yield similar values in both cases.

This issue can be resolved by taking the absolute value of the difference in fractional abundance between the two spectra as shown in Figure 2b. In the case of replicate results, the values obtained are small and yield an average value that is positive and close to zero. For isomeric data, significantly larger differences and higher averages are observed. Finally, we found that it is optimal to normalize absolute differences to the average value of the absolute fractional abundance of the fragments from both spectra. This normalization is rooted in the premise that the percent change in each peak height best reflects the contribution of each peak to differences in the overall spectrum and balances the influence of changes in intensity between small and large peaks. Normalized data are illustrated in Figure 2c for both isomer pairs and replicates.

In the case of isomeric data sets, some subset of fragments are likely to exhibit larger differences in fractional abundance than would be expected from replicate datasets due to the influence of the isomeric structures on the fragmentation process itself (the largest of these deviations would have previously been utilized in the *R*-value approach discussed above).^25^ All fragmentation channels that vary by more than the expected instrumental variation will cause the average difference in fractional abundance to be larger than that obtained from replicate experiments. The statistical likelihood that isomeric data differs from replicate measurements can be determined by calculating a *p*-value. Individual replicate and isomer difference datasets (where the replicate is simply a repeat experiment for one of the isomers) can be compared directly with a two-sample *t*-test. Alternatively, a more general approach employs a one-sample, one-tailed *t*-test against a hypothesized mean, which can be obtained from a series of replicate experiments collecting data from a variety of different peptides, as shown in Figure 2d. To implement this approach, we set the hypothesized mean for each dissociation method to be the average plus 3 standard deviations for each dataset shown in Figure 2d. This approach provides a conservatively high value for the hypothesized mean.

### Potential Factors That Influence Reproducibility

A number of instrument parameters could potentially influence the reproducibility of MS/MS spectra, ranging from voltages in the source region to resolution to various fragmentation parameters. If experiments are being conducted to identify isomers, all controllable parameters should be kept at identical values whenever possible. Furthermore, to offset the likelihood of influence from other factors typically outside instrument control (such as relative humidity or ambient temperature), experiments should be conducted on the same day or within the nearest temporal proximity that is feasible. However, to explore the likelihood that changes in instrumental parameters might yield false positives, we conducted replicate experiments where a range of experimental parameters were intentionally altered. The results are shown in Table 1. Although the average means are influenced by such changes, the differences are typically much smaller than those observed when isomeric spectra are compared. This allows conservative values to be used for the hypothesized mean, which greatly reduces the probability of false positives. On the other hand, if all conditions are strictly controlled and experiments conducted back-to-back, we note that our method could theoretically be used to identify changes in instrumental parameters if very strict hypothesized means were employed.

**Table 1:** CID Spectra Collected with Intentional Changes to Various Instrument Parameters^a^

	original parameter	changed parameter	observed mean
smaller isolation window	5 Da	1.5 Da	0.054
ion trap isolation	quadrupole isolation	ion trap isolation	0.019
lower RF%	150%	50%	0.019
lowering spray voltage	1700 V	1300 V	0.013
add source-induced dissociation	SID = 0 V	SID = 30 V	0.012
higher CID energy	CID = 23	CID = 30	0.079
**lower CID energy**	CID = 23	CID = 19	0.223
higher resolution	*R* = 60,000	*R* = 240,000	0.047

a The mean differences are relatively unaffected by most parameters except changing the collisional activation energy (bolded text).

The data in Figure 2 also make it clear that spectral reproducibility is dependent on the analyte itself. Although means from replicate experiments are all close to zero, they are not identical despite being individually reproducible. To use peptides as an example, the amino acid composition (including acidity/basicity, hydrophobicity, modifications, etc.), length, concentration, charge state, matrix (i.e., solvents, solvent ratios, other additives),^43^ and purity among others may all potentially influence the reproducibility of dissociation.

### Number of Scans and Statistics

To examine the influence of ion count on spectral reproducibility, we conducted experiments where we modified the automatic gain control to modulate the initial ion population. The results are shown in Figure S3, where ion count is plotted versus the observed mean (calculated as described above). Importantly, the mean exhibits a wide region of stability, comprising several orders of magnitude range of variation in the ion count before exceeding the means in Figure 2. These results suggest that analysis will not be extremely sensitive to variations in the source, but we suggest that ideally experiments be conducted with total ion count fluctuations within a factor of 10. The number of scans that are averaged for any dataset will also directly influence the size of the calculated mean. For direct infusion experiments, 50 scans or more are suggested (though typically we found no improvement over 100 scans). For LC–MS experiments, all available scans should be used, but the number of scans between comparisons must be equal, and the number of scans used to set the hypothesized mean must match the subsequent isomer comparisons.

**Figure 3:**
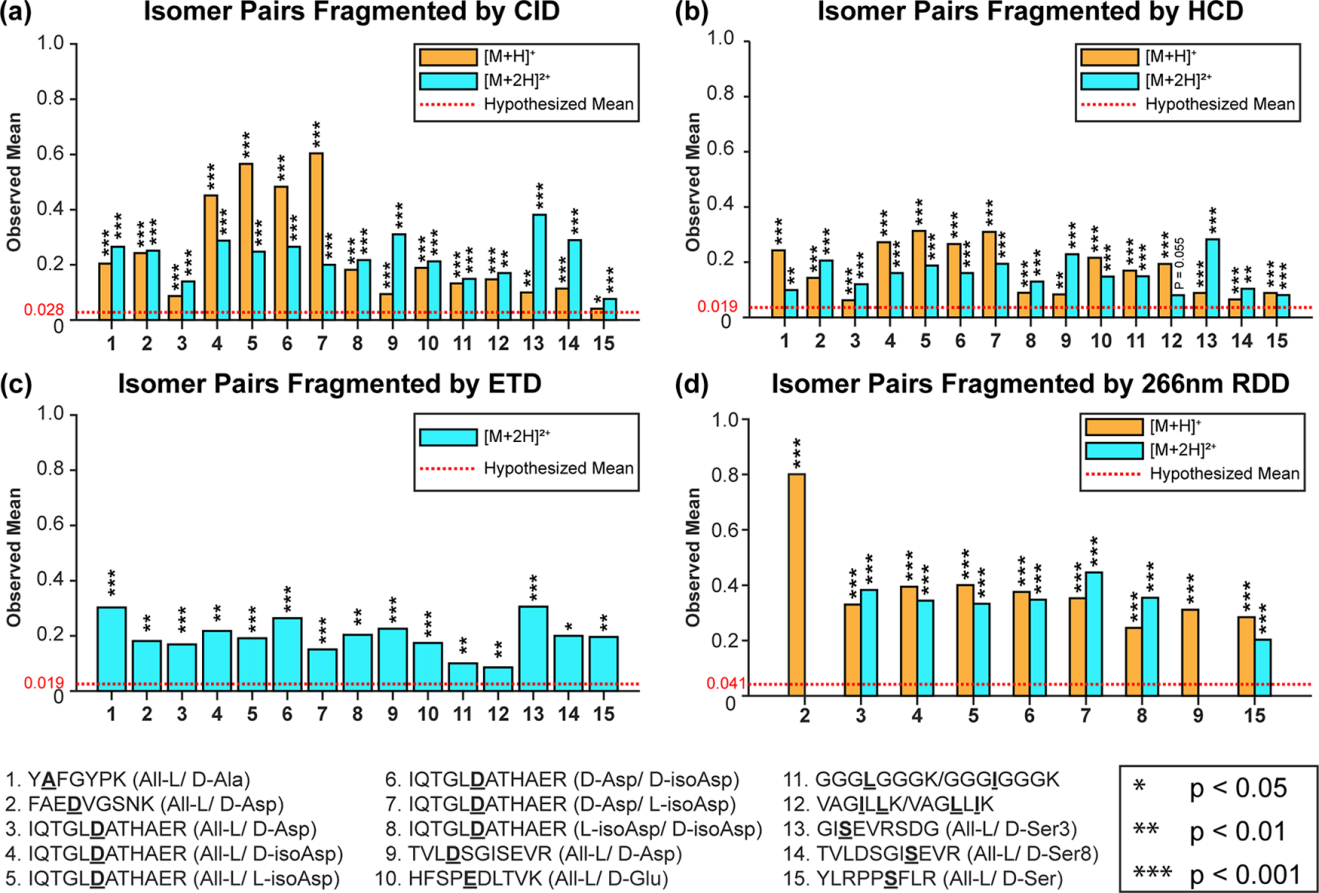
Calculated means for a variety of peptide isomers. *P*-values were determined by a one-sample *t*-test against a hypothesized mean (red dotted line) as described in the text. Results are binned by charge state and fragmentation method (a) CID, (b) HCD, (c) ETD, and (d) RDD. All isomer pairs can be differentiated by any fragmentation method (*p < 0.05, **p < 0.01, ***p < 0.001).

Having explored factors likely to influence our analysis, we next tested a variety of peptides with different fragmentation methods. The results are shown in Figure 3. Each bar represents the calculated mean for an isomeric peptide pair. The hypothesized mean, as determined from replicate experiments as described above, is shown by the red line for each MS/MS method. The *p*-value for the difference between the calculated mean and the hypothesized mean is indicated by stars above each bar. Importantly, many classes of isomers were examined within this set, including l/d alanine, which represents the smallest isomeric change that can be produced in a peptide. All four aspartic acid isomers, l/d glutamic acid, leucine/isoleucine, and l/d serine were also examined, representing some of the most common isomers encountered in biological samples. All of the dissociation methods are able to identify these isomers, and the probabilities that the data could be consistent with replicate results are generally very small (typically *p*-values < 0.001). Previous results based on *R*-value calculations have indicated that RDD provides the greatest structural sensitivity for isomer disambiguation by dissociation.^25^ The results in Figure 3 also show that RDD yields consistently high differences with the mean difference approach. CID also affords high selectivity for some peptides, although other peptides yield more similar spectra and with lower overall differences relative to replicates. Importantly, all peptides that we tested could be distinguished by CID. ETD also enables excellent differentiation, although the usual limitations in terms of suitable charge states will apply (i.e., +1 ions cannot be analyzed). Perhaps most surprising is the observation that HCD provides robust isomer identification despite the fact that the fragmentation spectra do not vary greatly between isomeric pairs (i.e., the observed mean differences are typically small. However, since HCD tends to yield fragment-rich spectra, the statistical confidence that small differences are robust is generally high. For one peptide pair, VAGILLK/VAGLLIK in the 2+ charge state, HCD could not distinguish them as isomers. This was the only failure we observed, and the same isomers were distinguishable by HCD in the 1+ charge state. Overall, the results in Figure 3 suggest that with direct infusion experiments, identification of isomeric pairs by mean difference should be highly robust.

**Figure 4:**
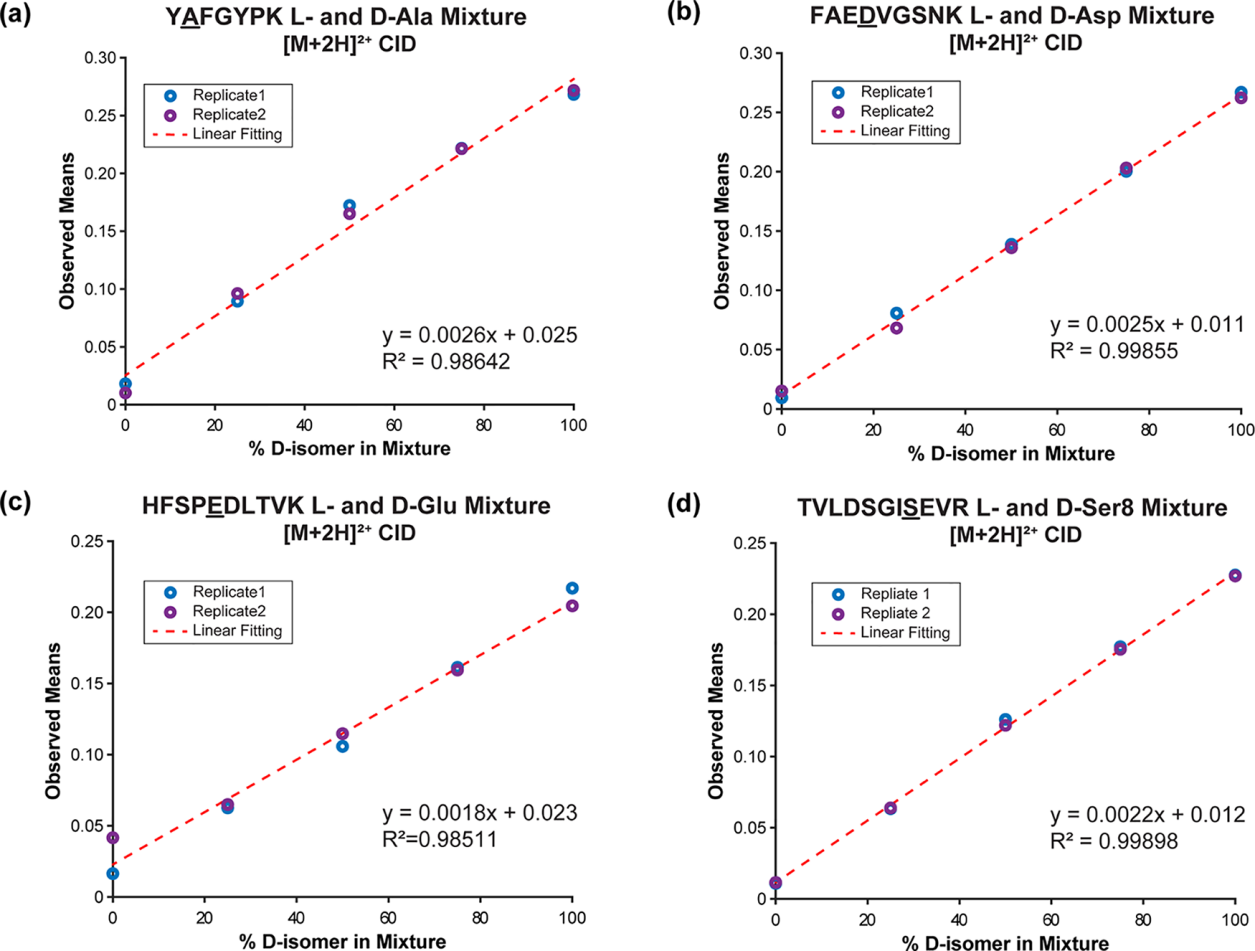
Calibration curves for observed means versus isomer composition for duplicate experiments (blue or purple circles). Highly linear results are observed for all 4 sets of peptide isomers: (a) YAFGYPK (l- and d-Ala), (b) FAEDVGSNK (l- and d-Asp), (c) HFSPEDLTVK (l- and d-Glu), and (d) TVLDSGISEVR (l- and d-Ser8).

The results in Figure 3 demonstrate that isolated isomers can easily be distinguished from each other by many MS/MS methods, but frequently isomeric species may be found in mixtures that are not easily separated. To evaluate whether our calculated means could be used to determine the fraction of each isomer present in a mixture, we collected data for calibration curves as shown in Figure 4. In each case, the plots of calculated mean versus percentage of one isomer yield highly linear fits. Furthermore, the slope of the lines relative to the reproducibility of replicate measurements suggests that compositions could be easily determined to within 5%. Previous attempts to use MS/MS data for calibration curves have generated non-linear results^28,44^ or required manipulation of the ratios to generate linear curves.^45^ The results in Figure 4 are further confirmation that mean differences in fragmentation spectra (calculated as described above) quantitatively reflect variation between isomeric peptides and that these differences are extremely reproducible.

### Application to LC–MS Data

**Figure 5:**
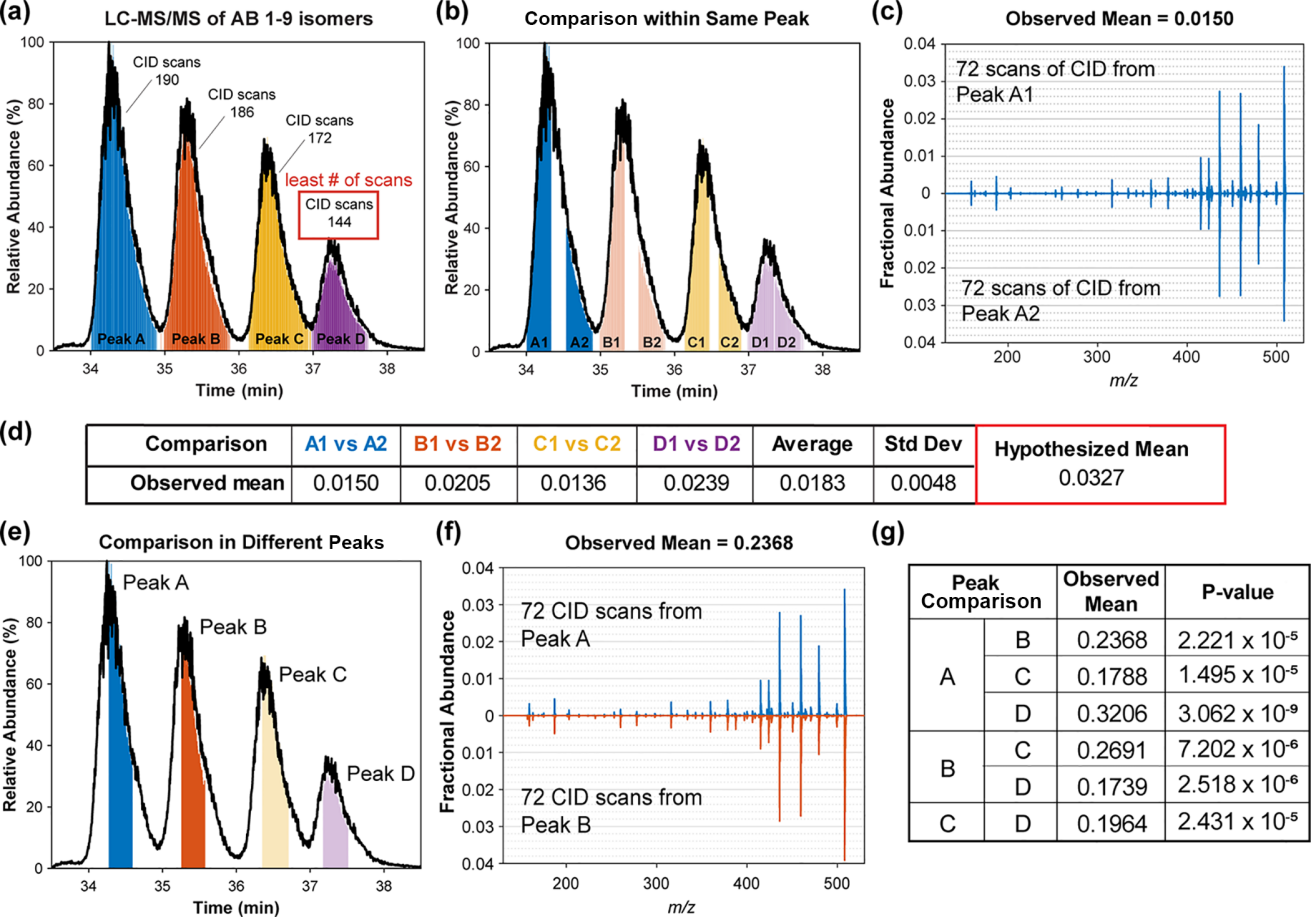
Workflow for identifying isomers with illustrative LC–MS data for Aβ(1–9) l- and l-isoAsp isomers. (a) Number of MS^2^ scans in each isomer dictates the scans required to determine the hypothesized value for statistical analysis. Peak D has the least number of scans (144 scans), (b) thus 72 scans would be extracted from both shoulders. (c) In the absence of coelution, the MS^2^ spectra are very similar and (d) can be used to determine the hypothesized mean for the data set. (e) 72 scans from the middle of each peak are compared against every other peak. (f) MS^2^ spectra from Peak A and Peak B, illustrating greater differences in fragmentation pattern. (g) Statistical analysis results are summarized and show that all 4 isomers can be identified.

Many aspects of an LC–MS assay differ from those employed for direct infusion experiments, including limited time for examining any given analyte, a solvent composition that can change with time, and the presence of competing ions. These differences require the recalculation of hypothesized means, as those determined by direct infusion will not be applicable. Furthermore, variations in the results between multiple LC–MS runs may be larger than differences between direct infusion experiments,^46,47^ which complicates the possibility for calculating a generic “LC-hypothesized mean”. Fortunately, given that LC–MS runs typically involve the analysis of complex mixtures, they should generally contain sufficient data to allow estimation of the hypothesized mean within a single dataset. To illustrate this concept, we collected LC–MS data from a simple mixture of Aβ(1–9) isomers (All-l, l-isoAsp1, l-isoAsp7, and l-isoAsp1 + l-isoAsp7). The chromatogram is shown in Figure 5a, where all four isomers are nicely resolved. To establish the hypothesized mean, we must determine the reproducibility of the CID spectra in this data. Each single peak is divided vertically, and the CID spectra from the leading and trailing edges are compared as illustrated in Figure 5b. Sample CID spectra from peak A are shown in Figure 5c. The calculated means from each single peak are used to generate the hypothesized mean as shown in the central table in Figure 5. This hypothesized mean can then be used to compare the central portion of each peak to the other isomeric forms as illustrated in Figure 5d. An example of the isomeric CID data is shown in Figure 5e. Finally, the results are summarized in Figure 5f, where it can be observed that all combinations of isomer peak comparisons yield high mean differences relative to replicates and low *p*-values.

**Figure 6:**
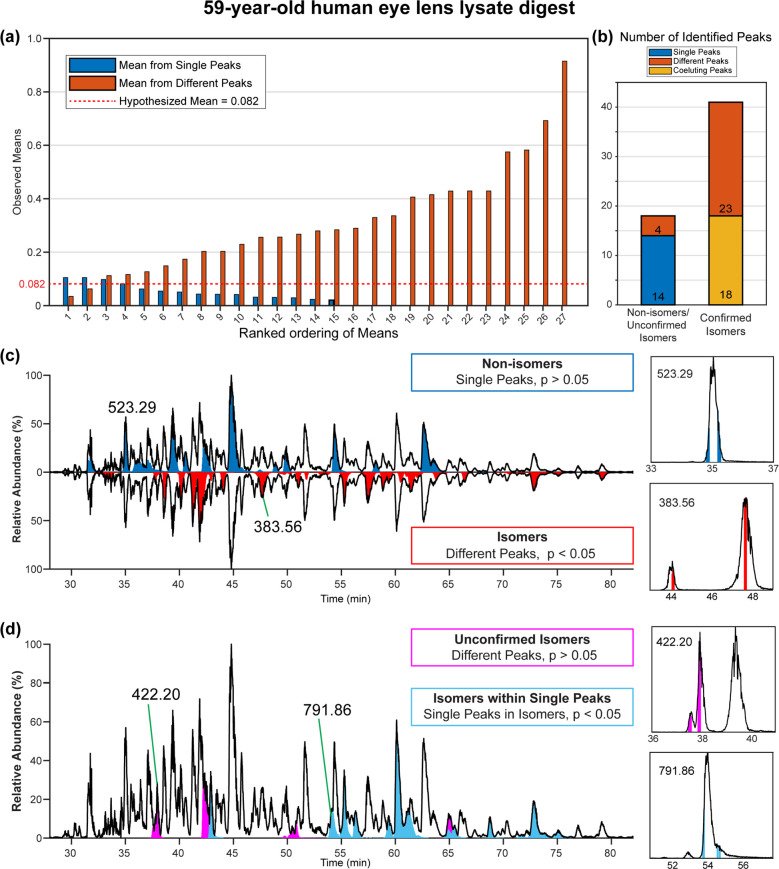
Tryptic digest of 59-year-old human eye lens crystallin with cataract. (a) Bar graph displays the calculated means for single peaks (blue) and for different peaks (orange). The hypothesized mean for this LC–MS/MS data was set to 0.06 (red dotted line) as described in the text. (b) Stacked bar graph showing the total numbers of assignments as a function of peak type. (c) LC chromatogram with non-isomers (*p* > 0.05) in blue and confirmed isomers in red (*p* < 0.05). (d) Unconfirmed isomers (different peaks, *p* > 0.05) are shown in pink and the isomers within coeluting peaks (*p* < 0.05) are shown in light blue. Examples of each type of peak comparison are on the right side of (b,c).

**Scheme 2:**
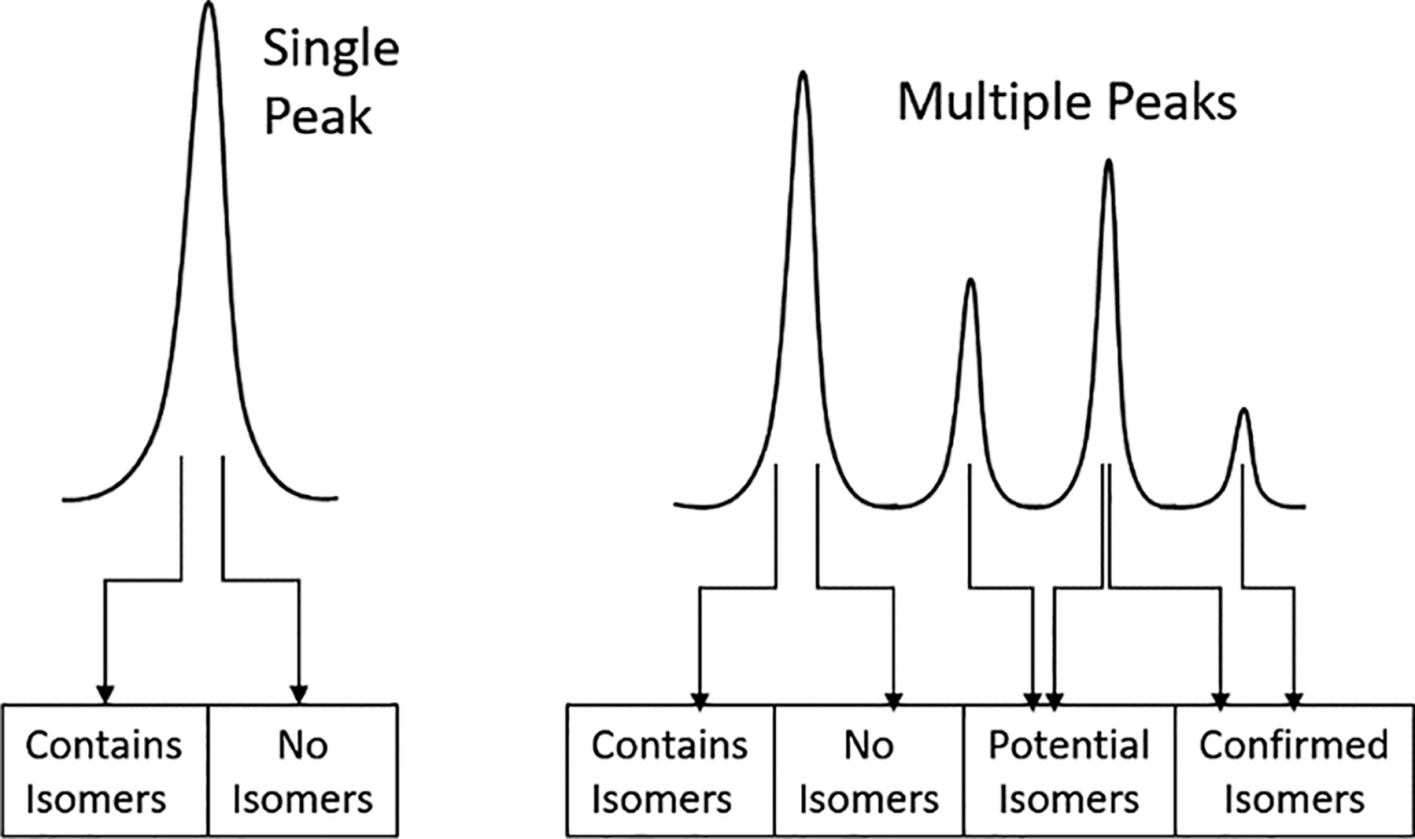
Potential Outcomes for LC–MS Data

A similar approach can be applied to more complex LC–MS data. Shown in Figure 6 are results from a tryptic digest of crystallin proteins from a human eye lens. In a “real-world” sample such as this, determination of the hypothesized mean requires additional consideration because the identity and location of isomers within the data are not known. First, identified peptides are checked for multiple elution times. If multiple peaks in the chromatogram are found, it is most likely that the peptide is isomerized and that each peak corresponds to at least one isomeric form. However, it is also possible that some isomers do not elute in discernably separate peaks, meaning that additional isomers may be hidden within single peaks (or within one peak of a group where some isomers were separated). These possibilities are outlined graphically in Scheme 2 for clarification.

To set the hypothesized mean difference, we calculated differences between all the multiple peaks (likely isomers) and compared those values to the mean differences from the leading and trailing edges of the single peaks (many of which are not likely to be isomers). The results are shown in Figure 6a. For the most part, the differences from single peaks are all lower than those observed for different peaks. This is consistent with most of the single peaks representing a single peptide. However, there are some individual peaks with means that are similar to some values obtained from different peaks. We therefore set the hypothesized mean difference to the upper quartile of the distribution for the single peak differences (0.08) for the purposes of statistical testing. The results after applying statistical testing to the entire dataset are shown in Figure 6b–d. Single peaks that do not likely contain isomers are shown in blue in Figure 6c, while isomers eluting in different peaks and confirmed by MS/MS data are shown in red. In Figure 6d, two additional classes are reported. A few peptides eluting in multiple peaks did not yield statistically significantly different CID spectra (pink peaks). Most likely, this occurs due to the reduced averaging and higher spectral variability inherent with LC–MS (relative to direct infusion), combined with low overall isomer discrimination capability of CID. It is possible that RDD or another fragmentation method might discriminate these isomers with LC–MS. However, CID was able to confirm the presence of several additional isomers that appeared to elute as single peaks, but where statistical comparison revealed differences across the elution profile.

## Conclusions

We have demonstrated that, surprisingly, isomeric peptides including examples of the most difficult cases can be easily distinguished by any MS/MS method by statistical comparison of differences in intensity among common fragment ions. It is clear that even subtle structural changes caused by isomerization are sufficient to alter the population of various dissociation channels well above typical reproducibility thresholds. This analysis can be implemented with both direct infusion and LC–MS experiments, and it is amenable to quantitation of binary mixtures. Although we have geared the present work toward analysis of isomers, the same method may be useful for comparing similar mass spectra in other contexts. For example, the method may be useful for normalizing collisional activation between instruments, comparing the similarity of common fragment ions in proteomic analyses, evaluating the influence of structure on dissociation, among many other possibilities. Furthermore, it is clear that with advances in modern instrumentation, even apparently subtle differences in mass spectra may be highly reproducible and contain important information.

## Supporting Information Available

Raw data extraction manual and additional experimental details, data, and spectra (PDF)

## References

[ref1] Kind T.; Tsugawa H.; Cajka T.; Ma Y.; Lai Z.; Mehta S. S.; Wohlgemuth G.; Barupal D. K.; Showalter M. R.; Arita M.; Fiehn O. Identification of Small Molecules Using Accurate Mass MS/MS Search. Mass Spectrom. Rev. 2018, 37, 513–532. 10.1002/mas.21535. 28436590 PMC8106966

[ref2] Wörner T. P.; Shamorkina T. M.; Snijder J.; Heck A. J. R. Mass Spectrometry-Based Structural Virology. Anal. Chem. 2021, 93, 620–640. 10.1021/acs.analchem.0c04339. 33275424 PMC7807421

[ref3] Abdulbagi M.; Wang L.; Siddig O.; Di B.; Li B. D-Amino Acids and D-Amino Acid-Containing Peptides: Potential Disease Biomarkers and Therapeutic Targets?. Biomolecules 2021, 11, 1716 10.3390/biom11111716. 34827714 PMC8615943

[ref4] Lambeth T. R.; Riggs D. L.; Talbert L. E.; Tang J.; Coburn E.; Kang A. S.; Noll J.; Augello C.; Ford B. D.; Julian R. R. Spontaneous Isomerization of Long-Lived Proteins Provides a Molecular Mechanism for the Lysosomal Failure Observed in Alzheimer’s Disease. ACS Cent. Sci. 2019, 5, 1387–1395. 10.1021/acscentsci.9b00369. 31482121 PMC6716341

[ref5] Hubbard E. E.; Heil L. R.; Merrihew G. E.; Chhatwal J. P.; Farlow M. R.; McLean C. A.; Ghetti B.; Newell K. L.; Frosch M. P.; Bateman R. J.; Larson E. B.; Keene C. D.; Perrin R. J.; Montine T. J.; MacCoss M. J.; Julian R. R. Does Data-Independent Acquisition Data Contain Hidden Gems? A Case Study Related to Alzheimer’s Disease. J. Proteome Res. 2022, 21, 118–131. 10.1021/acs.jproteome.1c00558. 34818016 PMC8741752

[ref6] Watanabe A.; Takio K.; Ihara Y. Deamidation and Isoaspartate Formation in Smeared Tau in Paired Helical Filaments. J. Biol. Chem. 1999, 274, 7368–7378. 10.1074/jbc.274.11.7368. 10066801

[ref7] Peng W.; Goli M.; Mirzaei P.; Mechref Y. Revealing the Biological Attributes of N-Glycan Isomers in Breast Cancer Brain Metastasis Using Porous Graphitic Carbon (PGC) Liquid Chromatography-Tandem Mass Spectrometry (LC-MS/MS). J. Proteome Res. 2019, 18, 3731–3740. 10.1021/acs.jproteome.9b00429. 31430160

[ref8] Kyle J. E.; Zhang X.; Weitz K. K.; Monroe M. E.; Ibrahim Y. M.; Moore R. J.; Cha J.; Sun X.; Lovelace E. S.; Wagoner J.; Polyak S. J.; Metz T. O.; Dey S. K.; Smith R. D.; Burnum-Johnson K. E.; Baker E. S. Uncovering Biologically Significant Lipid Isomers with Liquid Chromatography, Ion Mobility Spectrometry and Mass Spectrometry. Analyst 2016, 141, 1649–1659. 10.1039/C5AN02062J. 26734689 PMC4764491

[ref9] Idres N.; Marill J.; Flexor M. A.; Chabot G. G. Activation of Retinoic Acid Receptor-Dependent Transcription by All-Trans-Retinoic Acid Metabolites and Isomers. J. Biol. Chem. 2002, 277, 31491–31498. 10.1074/jbc.M205016200. 12070176

[ref10] Fa M.; Diana A.; Carta G.; Cordeddu L.; Melis M.; Murru E.; Sogos V.; Banni S. Incorporation and Metabolism of C9,T11 and T10,C12 Conjugated Linoleic Acid (CLA) Isomers in Rat Brain. Biochim. Biophys. Acta, Mol. Cell Biol. Lipids 2005, 1736, 61–66. 10.1016/j.bbalip.2005.06.010. 16055372

[ref11] Manik M. K.; Shi Y.; Li S.; Zaydman M. A.; Damaraju N.; Eastman S.; Smith T. G.; Gu W.; Masic V.; Mosaiab T.; Weagley J. S.; Hancock S. J.; Vasquez E.; Hartley-Tassell L.; Kargios N.; Maruta N.; Lim B. Y. J.; Burdett H.; Landsberg M. J.; Schembri M. A.; Prokes I.; Song L.; Grant M.; DiAntonio A.; Nanson J. D.; Guo M.; Milbrandt J.; Ve T.; Kobe B. Cyclic ADP Ribose Isomers: Production, Chemical Structures, and Immune Signaling. Science 2022, 377, eadc8969 10.1126/science.adc8969. 36048923 PMC13025242

[ref12] National Research Council (US); Committee on Assessing the Importance and Impact of Glycomics and Glycosciences. In Transforming Glycoscience: A Roadmap for the Future; National Academies Press, 2012. 23270009

[ref13] Edwards H. M.; Wu H. T.; Julian R. R.; Jackson G. P. Differentiating Aspartic Acid Isomers and Epimers with Charge Transfer Dissociation Mass Spectrometry (CTD-MS). Analyst 2022, 147, 1159–1168. 10.1039/D1AN02279B. 35188507

[ref14] Scriba G. K. E. Recent Advances in Enantioseparations of Peptides by Capillary Electrophoresis. Electrophoresis 2003, 24, 4063–4077. 10.1002/elps.200305657. 14661234

[ref15] Kirschbaum C.; Saied E. M.; Greis K.; Mucha E.; Gewinner S.; Schöllkopf W.; Meijer G.; Helden G.; Poad B. L. J.; Blanksby S. J.; Arenz C.; Pagel K. Resolving Sphingolipid Isomers Using Cryogenic Infrared Spectroscopy. Angew. Chem., Int. Ed. 2020, 59, 13638–13642. 10.1002/anie.202002459. PMC7496694 32291895

[ref16] Wu H.-T.; Julian R. R. Two-Dimensional Identification and Localization of Isomers in Crystallin Peptides Using TWIM-MS. Analyst 2020, 145, 5232–5241. 10.1039/D0AN01036G. 32608408

[ref17] Van Orman B. L.; Wu H.-T.; Julian R. R. Differentiation of Peptide Isomers by Excited-State Photodissociation and Ion–Molecule Interactions. Phys. Chem. Chem. Phys. 2020, 22, 23678–23685. 10.1039/D0CP04111D. 33052992

[ref18] Ashline D. J.; Lapadula A. J.; Liu Y.-H.; Lin M.; Grace M.; Pramanik B.; Reinhold V. N. Carbohydrate Structural Isomers Analyzed by Sequential Mass Spectrometry. Anal. Chem. 2007, 79, 3830–3842. 10.1021/ac062383a. 17397137 PMC3064989

[ref19] Young R. S. E.; Flakelar C. L.; Narreddula V. R.; Jekimovs L. J.; Menzel J. P.; Poad B. L. J.; Blanksby S. J. Identification of Carbon-Carbon Double Bond Stereochemistry in Unsaturated Fatty Acids by Charge-Remote Fragmentation of Fixed-Charge Derivatives. Anal. Chem. 2022, 94, 16180–16188. 10.1021/acs.analchem.2c03625. 36342869

[ref20] EdwardsH. M.; WuH.; JulianR. R.; JacksonG. P. Differentiation of Leucine and Isoleucine Residues in Peptides Using Charge Transfer Dissociation Mass Spectrometry (CTD-MS). Rapid Commun. Mass Spectrom. 2022, 36, e924610.1002/rcm.9246.34927767

[ref21] Bai L.; Romanova E. V.; Sweedler J. V. Distinguishing Endogenous D-Amino Acid-Containing Neuropeptides in Individual Neurons Using Tandem Mass Spectrometry. Anal. Chem. 2011, 83, 2794–2800. 10.1021/ac200142m. 21388150 PMC3077102

[ref22] Adams C. M.; Zubarev R. A. Distinguishing and Quantifying Peptides and Proteins Containing D-Amino Acids by Tandem Mass Spectrometry. Anal. Chem. 2005, 77, 4571–4580. 10.1021/ac0503963. 16013875

[ref23] Pham H. T.; Julian R. R. Characterization of Glycosphingolipid Epimers by Radical-Directed Dissociation Mass Spectrometry. Analyst 2016, 141, 1273–1278. 10.1039/C5AN02383A. 26800360

[ref24] Pham H. T.; Ly T.; Trevitt A. J.; Mitchell T. W.; Blanksby S. J. Differentiation of Complex Lipid Isomers by Radical-Directed Dissociation Mass Spectrometry. Anal. Chem. 2012, 84, 7525–7532. 10.1021/ac301652a. 22881372

[ref25] Zhang X.; Julian R. R. Radical Mediated Dissection of Oligosaccharides. Int. J. Mass Spectrom. 2014, 372, 22–28. 10.1016/j.ijms.2014.07.045.

[ref26] Tao Y.; Quebbemann N. R.; Julian R. R. Discriminating D-Amino Acid-Containing Peptide Epimers by Radical-Directed Dissociation Mass Spectrometry. Anal. Chem. 2012, 84, 6814–6820. 10.1021/ac3013434. 22812429

[ref27] Lambeth T. R.; Julian R. R. Efficient Isothiocyanate Modification of Peptides Facilitates Structural Analysis by Radical-Directed Dissociation. J. Am. Soc. Mass Spectrom. 2022, 33, 1338–1345. 10.1021/jasms.1c00237. 34670075

[ref28] Hui J. O.; Flick T.; Loo J. A.; Campuzano I. D. G. Unequivocal Identification of Aspartic Acid and IsoAspartic Acid by MALDI-TOF/TOF: From Peptide Standards to a Therapeutic Antibody. J. Am. Soc. Mass Spectrom. 2021, 32, 1901–1909. 10.1021/jasms.0c00370. 33390012

[ref29] Nagy G.; Pohl N. L. B. Complete Hexose Isomer Identification with Mass Spectrometry. J. Am. Soc. Mass Spectrom. 2015, 26, 677–685. 10.1007/s13361-014-1072-z. 25652933

[ref30] Pesyna G. M.; Venkataraghavan R.; Dayringer H. E.; McLafferty F. W. Probability Based Matching System Using a Large Collection of Reference Mass Spectra. Anal. Chem. 1976, 48, 1362–1368. 10.1021/ac50003a026.

[ref31] Liu J.; Bell A. W.; Bergeron J. J.; Yanofsky C. M.; Carrillo B.; Beaudrie C. E.; Kearney R. E. Methods for Peptide Identification by Spectral Comparison. Proteome Sci. 2007, 5, 3 10.1186/1477-5956-5-3. 17227583 PMC1783643

[ref32] Eng J. K.; Fischer B.; Grossmann J.; MacCoss M. J. A Fast SEQUEST Cross Correlation Algorithm. J. Proteome Res. 2008, 7, 4598–4602. 10.1021/pr800420s. 18774840

[ref33] Frank A. M. Predicting Intensity Ranks of Peptide Fragment Ions. J. Proteome Res. 2009, 8, 2226–2240. 10.1021/pr800677f. 19256476 PMC2738854

[ref34] Dorfer V.; Pichler P.; Stranzl T.; Stadlmann J.; Taus T.; Winkler S.; Mechtler K. MS Amanda, a Universal Identification Algorithm Optimized for High Accuracy Tandem Mass Spectra. J. Proteome Res. 2014, 13, 3679–3684. 10.1021/pr500202e. 24909410 PMC4119474

[ref35] Demuth W.; Karlovits M.; Varmuza K. Spectral Similarity versus Structural Similarity: Mass Spectrometry. Anal. Chim. Acta 2004, 516, 75–85. 10.1016/j.aca.2004.04.014.

[ref36] Yilmaz Ş.; Vandermarliere E.; Martens L. Methods to Calculate Spectrum Similarity. In Proteome Bioinformatics; Keerthikumar S.; Mathivanan S., Eds.; Methods in Molecular Biology; Humana Press, 2017; Vol. 1549, pp 75–100. 10.1007/978-1-4939-6740-7_7 27975285

[ref37] Frewen B. E.; Merrihew G. E.; Wu C. C.; Noble W. S.; MacCoss M. J. Analysis of Peptide MS/MS Spectra from Large-Scale Proteomics Experiments Using Spectrum Libraries. Anal. Chem. 2006, 78, 5678–5684. 10.1021/ac060279n. 16906711

[ref38] Wan K. X.; Vidavsky I.; Gross M. L. Comparing Similar Spectra: From Similarity Index to Spectral Contrast Angle. J. Am. Soc. Mass Spectrom. 2002, 13, 85–88. 10.1016/S1044-0305(01)00327-0. 11777203

[ref39] Hope J. L.; Sinha A. E.; Prazen B. J.; Synovec R. E. Evaluation of the DotMap Algorithm for Locating Analytes of Interest Based on Mass Spectral Similarity in Data Collected Using Comprehensive Two-Dimensional Gas Chromatography Coupled with Time-of-Flight Mass Spectrometry. J. Chromatogr. A 2005, 1086, 185–192. 10.1016/j.chroma.2005.06.026. 16130672

[ref40] Hood C. A.; Fuentes G.; Patel H.; Page K.; Menakuru M.; Park J. H. Fast Conventional Fmoc Solid-Phase Peptide Synthesis with HCTU. J. Pept. Sci. 2008, 14, 97–101. 10.1002/psc.921. 17890639

[ref41] Lyon Y. A.; Sabbah G. M.; Julian R. R. Differences in α-Crystallin Isomerization Reveal the Activity of Protein Isoaspartyl Methyltransferase (PIMT) in the Nucleus and Cortex of Human Lenses. Exp. Eye Res. 2018, 171, 131–141. 10.1016/j.exer.2018.03.018. 29571628 PMC5964019

[ref42] Armentrout P. B. Energetics and Mechanisms for Decomposition of Cationized Amino Acids and Peptides Explored Using Guided Ion Beam Tandem Mass Spectrometry. Mass Spectrom. Rev. 2021, 42, 928–953. 10.1002/mas.21723. 34392555

[ref43] Trufelli H.; Palma P.; Famiglini G.; Cappiello A. An Overview of Matrix Effects in Liquid Chromatography–Mass Spectrometry. Mass Spectrom. Rev. 2011, 30, 491–509. 10.1002/mas.20298. 21500246

[ref44] Indeykina M. I.; Popov I. A.; Kozin S. A.; Kononikhin A. S.; Kharybin O. N.; Tsvetkov P. O.; Makarov A. A.; Nikolaev E. N. Capabilities of MS for Analytical Quantitative Determination of the Ratio of α- and ΒAsp7 Isoforms of the Amyloid-β Peptide in Binary Mixtures. Anal. Chem. 2011, 83, 3205–3210. 10.1021/ac103213j. 21410227

[ref45] Riggs D. L.; Silzel J. W.; Lyon Y. A.; Kang A. S.; Julian R. R. Analysis of Glutamine Deamidation: Products, Pathways, and Kinetics. Anal. Chem. 2019, 91, 13032–13038. 10.1021/acs.analchem.9b03127. 31498611 PMC8805438

[ref46] Kalli A.; Hess S. Effect of Mass Spectrometric Parameters on Peptide and Protein Identification Rates for Shotgun Proteomic Experiments on an LTQ-Orbitrap Mass Analyzer. Proteomics 2012, 12, 21–31. 10.1002/pmic.201100464. 22065615

[ref47] Tabb D. L.; Vega-Montoto L.; Rudnick P. A.; Variyath A. M.; Ham A.-J. L.; Bunk D. M.; Kilpatrick L. E.; Billheimer D. D.; Blackman R. K.; Cardasis H. L.; Carr S. A.; Clauser K. R.; Jaffe J. D.; Kowalski K. A.; Neubert T. A.; Regnier F. E.; Schilling B.; Tegeler T. J.; Wang M.; Wang P.; Whiteaker J. R.; Zimmerman L. J.; Fisher S. J.; Gibson B. W.; Kinsinger C. R.; Mesri M.; Rodriguez H.; Stein S. E.; Tempst P.; Paulovich A. G.; Liebler D. C.; Spiegelman C. Repeatability and Reproducibility in Proteomic Identifications by Liquid Chromatography–Tandem Mass Spectrometry. J. Proteome Res. 2010, 9, 761–776. 10.1021/pr9006365. 19921851 PMC2818771

