# Supplementary material for: Statistical Framework for Identifying Differences in Similar Mass Spectra: Expanding Possibilities for Isomer Identification
Source: Anal Chem. 2023 Apr 17;95(17):6996–7005. doi: 10.1021/acs.analchem.3c00495 (PMC10157605; doi:10.1021/acs.analchem.3c00495)
Supplement: Supplementary file 1 — ac3c00495_si_001.pdf [file ac3c00495_si_001.pdf]

SUPPORTING INFORMATION

A statistical framework for identifying differences in similar mass spectra: Expanding possibilities for isomer identification

Hoi Ting Wu, Dylan L. Riggs, Yana A. Lyon, and Ryan R. Julian*

Department of Chemistry, University of California, Riverside, California 92521, United States

* Corresponding author: Ryan R. Julian

E-mail: ryan.julian@ucr.edu

Table of Content

User manual for Isomer Difference Software ……………………………………………..…….. S2

Figure S3. Total ion count vs observed mean. ……………………………………….……..... S4

Figure S4-6. Extracted chromatograms from 59-year-old human eye lens. ……..….….….... S5-S7

Figure S7. Isomer differentiation with Mahalanobis Distance …………………………………… S8

References ………………………………………………………………………………………. S9

User manual for Isomer Difference Software

The Julian Lab develops programs that extract the m/z and the ion count of common ions between two spectra from direction infusion or liquid chromatography data. Thermo raw data can be imported directly onto both programs. The software is available on request from the authors.

Isomer Difference Software is used for direct infusion data. Simply, the files are imported onto the program using “Load File 1” and “Load File 2”. Depending on the detector resolution, the mass resolution can be modified. For high resolution data (R = 30,000), Mass Resolution is set to 5 ppm. The “Precursor Mass” input allows the users to exclude the data at that mass range with “Precursor width”. The “# Peaks to Average” extracts the number of data user defines. Once the setup is complete, “Process Spectra” will output the common ions m/z and its ion count.

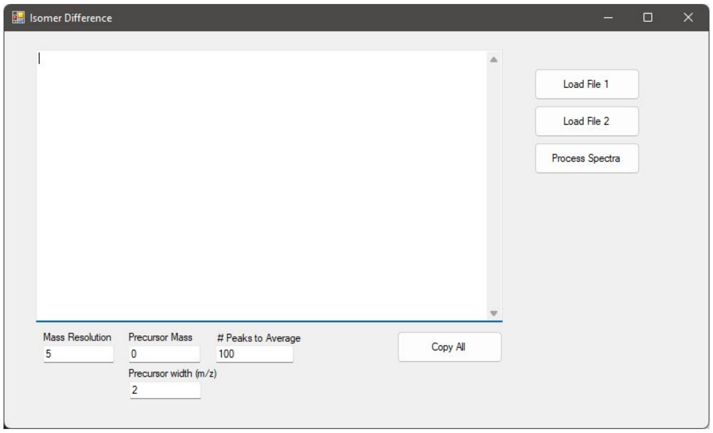

Figure S1. Software interface of the Isomer Difference Software.

LCMSreader Software is used for LC-MS data. Simply, the files are imported onto the program using “open file 1” Depending on the detector resolution, the mass resolution can be modified. Since LC-MS signals verify more, mass resolution is set to 20 ppm. The “Precursor Mass” input allows the users to exclude the data at that mass range. The first and last desired spectra scan numbers will be listed in “firstscan” and “lastscan” for both regions, which the program extracts the common ions m/z and ion count in the input scans ranch. Once the setup is complete, “Process Spectra” will output the common ions m/z and its ion count.

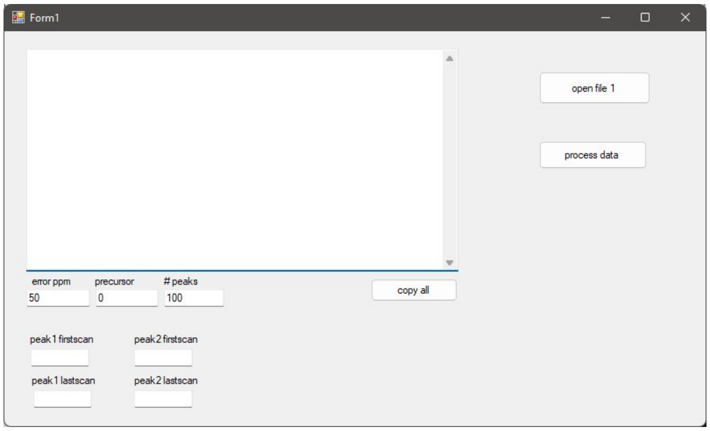

Figure S2. Software interface of the LCMSReader Software.

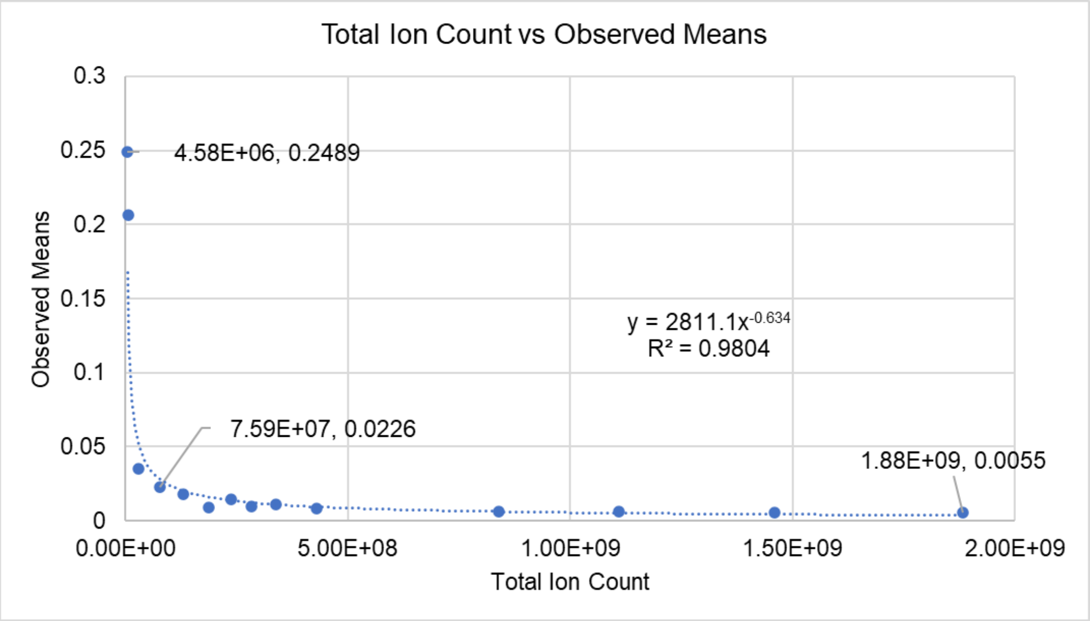

Figure S3. The observed mean was found at various total ion count (TIC) level. The observed means appear to be stabilized from a few orders of magnitude in TIC range.

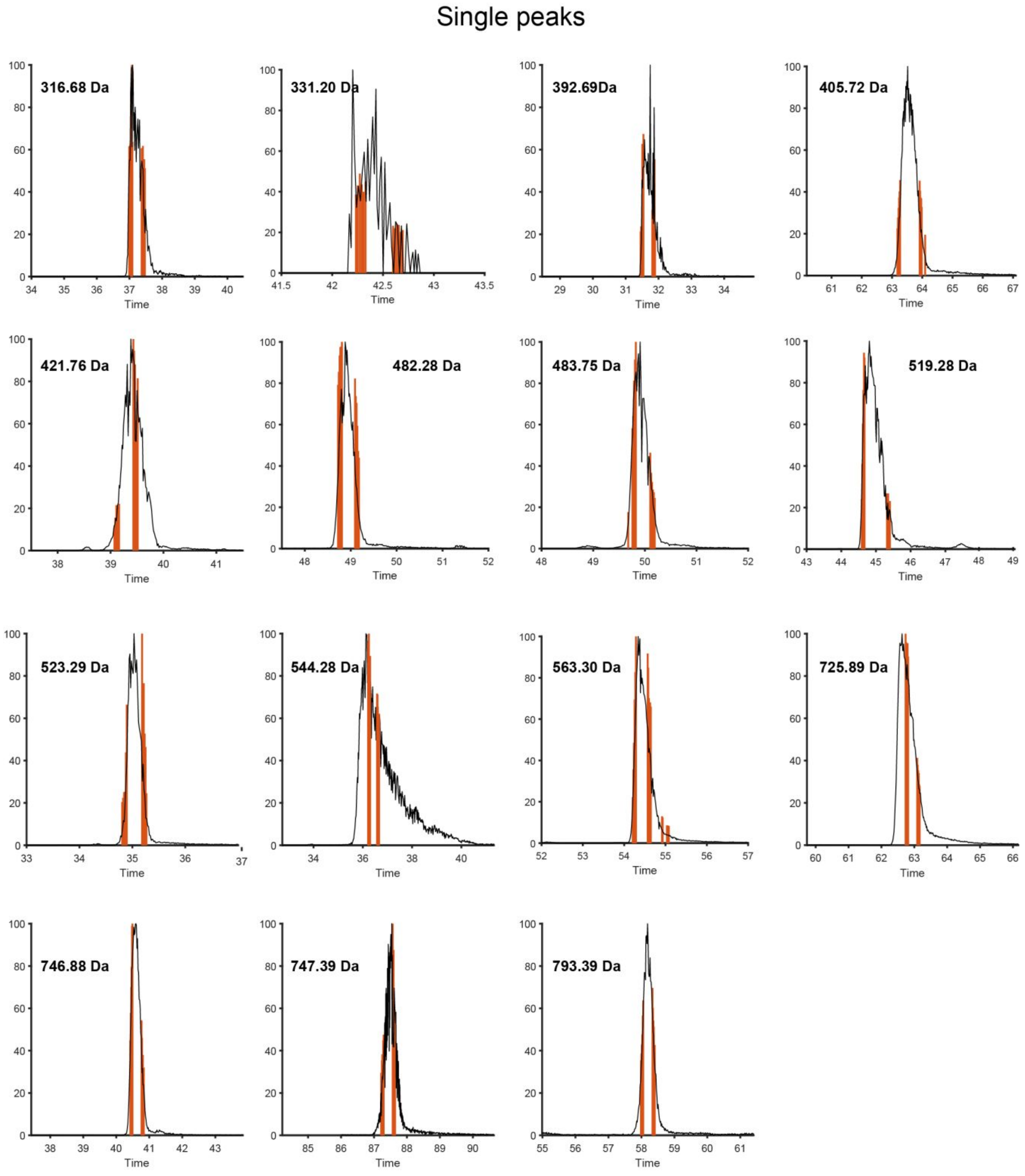

Figure S4. Extracted chromatograms from 59-year-old human eye lens lysate digest identified as “Single Peaks” are shown. The orange bars represent the CID scans available and used to compare the fragmentation patterns.

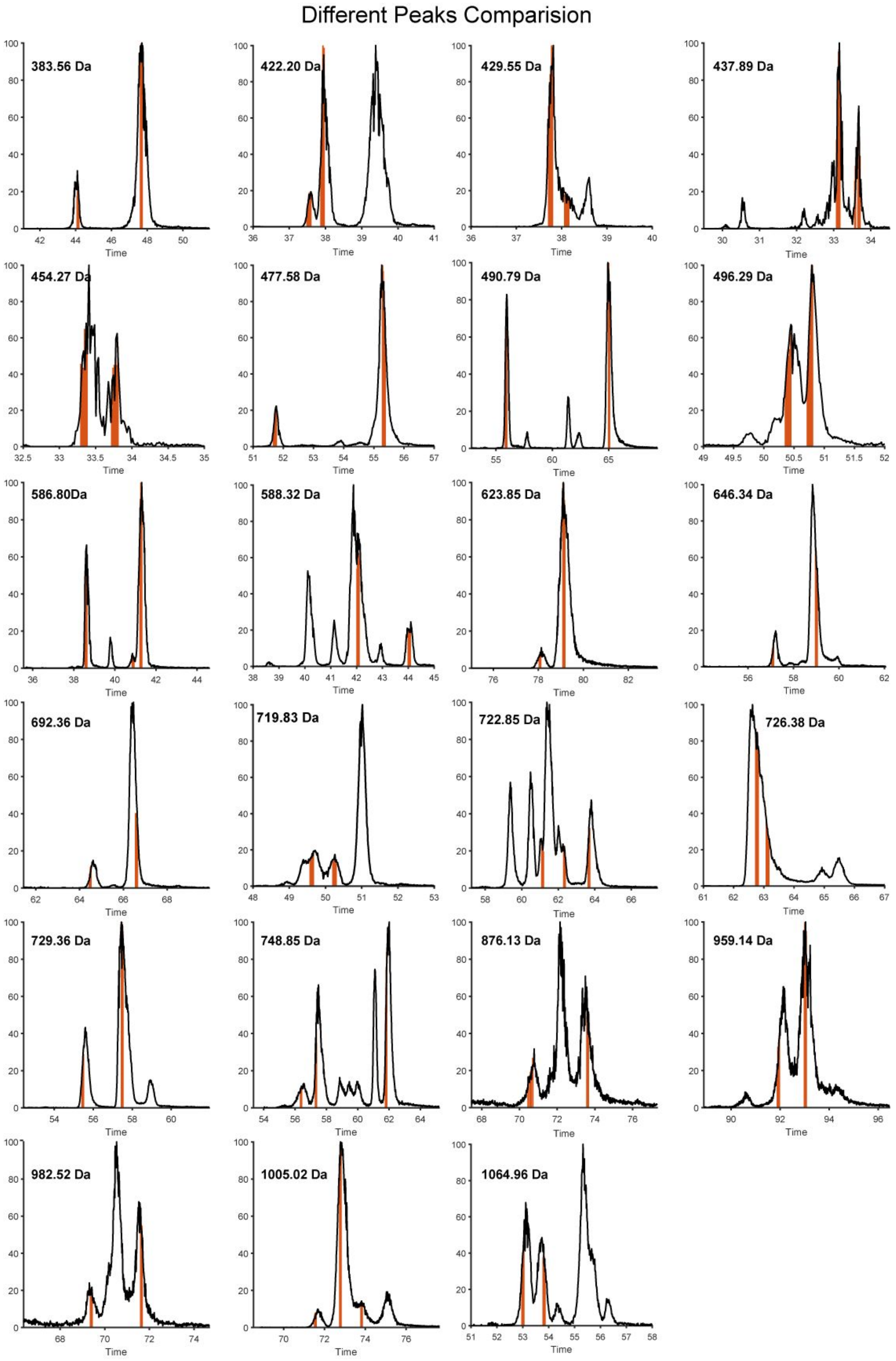

Figure S5. Extracted chromatograms from 59-year-old human eye lens lysate digest identified as “Different Peaks” are shown. The orange bars represent the CID scans available and used to compare the fragmentation patterns.

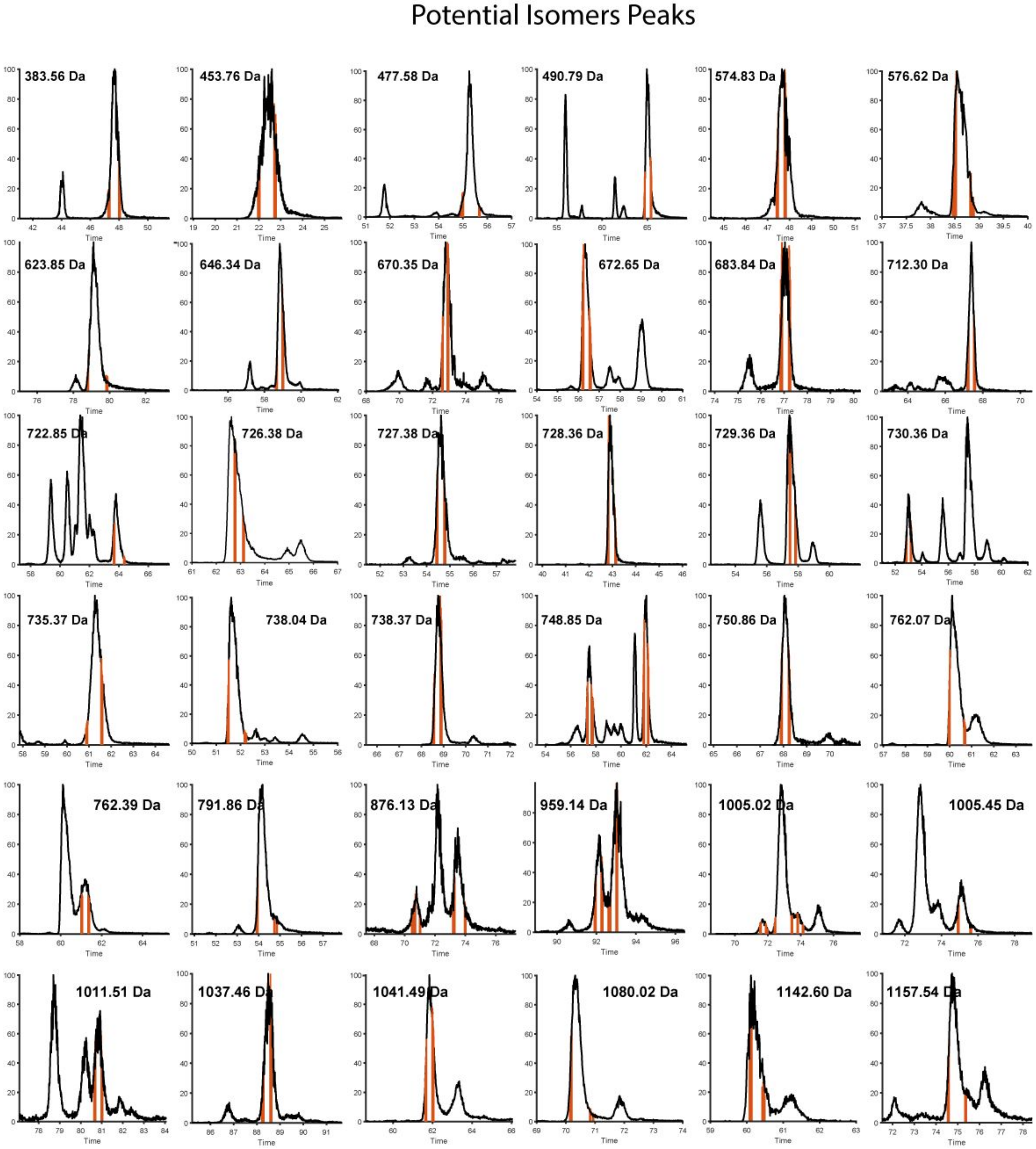

Figure S6. Extracted chromatograms from 59-year-old human eye lens lysate digest identified as "Potential Isomers" are shown. The orange bars represent the CID scans available and used to compare the fragmentation patterns.

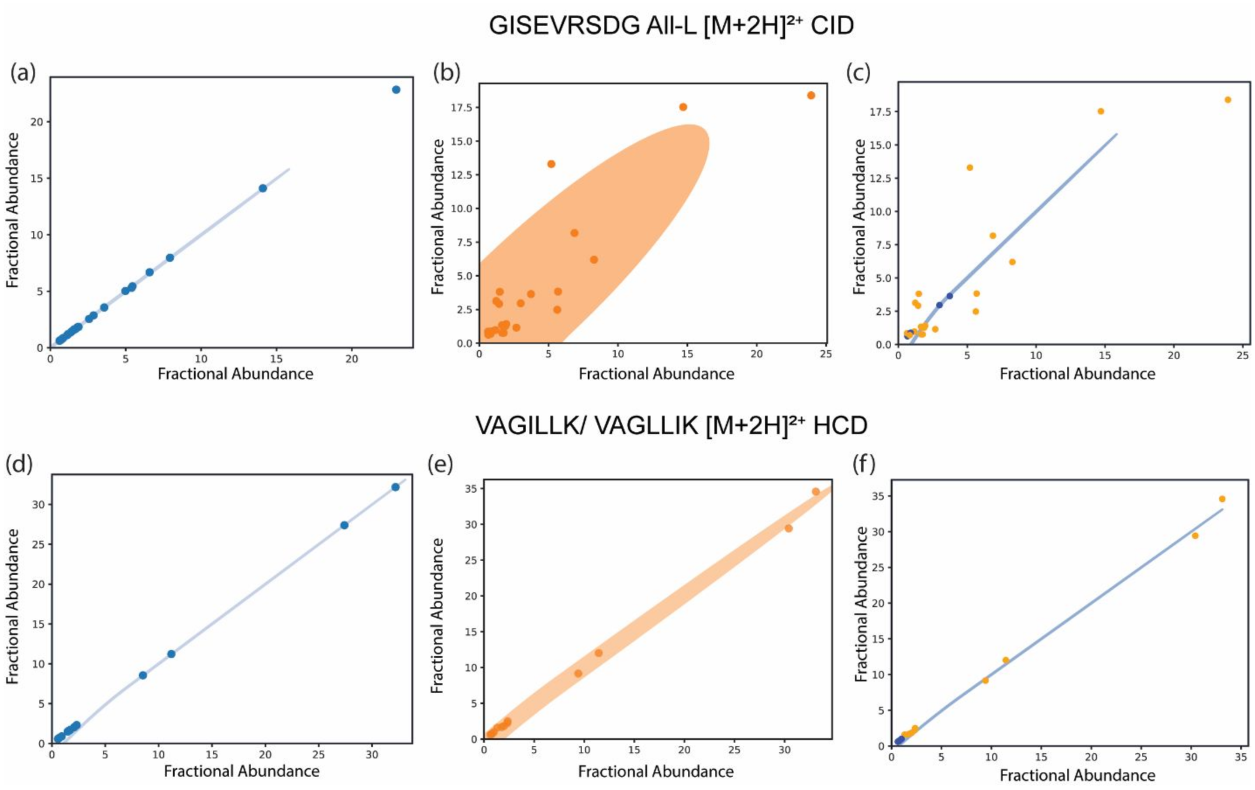

Figure S7. Isomer differentiation with Mahalanobis Distance for **(a-c)** GISEVRSDG 2+ (All-L vs D-Ser3) fragmented by CID and **(d-e)** VAGILLK/ VAGLLIK 2+ fragmented by HCD. In **(a, d),** the fractional abundances for replicates are plotted as blue circles, while in **(b, e)** peptide isomers are orange. Shaded regions indicate 95% probability zones based on the observed data points. In **(c, f)**, the 95% prediction ellipse derived from replicate data is plotted with individual isomer data points. Isomer data points within the ellipse are shown as dark blue circles, whereas outliers are shown in light orange circles.

The results in Fig. S7 show that variation in fractional abundance is greater when comparing isomers than for replicates. If replicate data is used as the reference population, most isomeric data points fall outside the 95% predicted area. However, there are potential complications with this approach. For example, the replicate data for both peptides produce nearly linear correlation, which can be problematic for the calculation of Mahalanobis distances. When two variables are highly correlated, the inverse matrix (required to calculate the Mahalanobis distance) may not exist or may be unreliable.[1]

References

[1] Varmuza, K.; Filzmoser, P. Multivariate Data. In *Introduction to Multivariate Statistical Analysis in Chemometrics*; CRC Press: Boca Raton, 2009; pp 31–58.
